# Complicated renal colic: usefulness of untrasonography in Emergency Department

**DOI:** 10.1186/2036-7902-4-S1-A28

**Published:** 2012-12-18

**Authors:** Alberto Oviedo-García, M Algaba-Montes, J Lopez-Libano, JM Alvarez-Franco, N Diaz-Rodriguez, A Rodriguez-Lorenzo

**Affiliations:** 1 Emergency Department, Valme Hospital, Seville, Members of the Working Group of ultrasound SEMES_Andalucía and Semergen, Spain; 2 Critical Care Department, Miramar Hospital, Mallorca, Member of the Working Group of ultrasound Semergen, Spain; 3 Emergency Department, IB-Salut, Ibiza, Member of the Working Group of ultrasound Semergen, Spain; 4 Primary Care, Barbadás Primary Care Center, Ourense, Member of the Working Group of ultrasound Semergen, Spain; 5 Radiology Department, Perpetuo Socorro Hospital, Vigo, Member of the Working Group of ultrasound Semergen, Spain

## Background

The renal colic (RC) is a common urological emergency, very painful, requiring prompt diagnosis and treatment. Over 12% of the population will experience a RC during their life, with the rate of recurrence by about 50%. It is caused by an acute ureteral obstruction, which in most cases is usually due to a lithiasis, causing acute distension of the collecting system. Urinoma is an uncommon disease, caused by the extravasation of urine from any constituent of the urinary tract (kidney, ureter, urinary bladder or the urethra); it may be confined, encapsulated fluid collections or may manifest as free fluid.

## Objetive

Abdominal ultrasound training in emergency department (ED).

## Patients and methods

We present the diagnosis of a RC with urinoma by ultrasound assessment in ED. We used a Sonosite M-Turbo ultrasound with Convex probe C60 2-5 MHz and abdominal ultrasound software.

## Results

Abdominal ultrasound is a noninvasive, fast, portable, repeatable, and inexpensive method that gives us information on the degree of kidney affection pelvicalyceal ectasia can also diagnose complications such as urinoma. Urinoma is defined as an encapsulated collection of extravasated urine in the perirenal or paraureteral space, favored by the presence of an obstruction, provided that renal function is maintained. The most common causes of urinoma include obstructive uropathy, abdominal trauma with involvement of the urinary tract, inflammatory/malignant retroperitoneal, lithotripsy, nephrostomy or nonfunctioning kidney biopsy. One of the problems urinoma is the possibility of infection, thus complicating the development and may require surgery.

## Conclusion

Incorporating ultrasound Health Centers could facilitate early diagnosis of complications of CN, avoiding serious consequences, providing to the patient more clinic safety.
